# A Spectral Probe for Detection of Aluminum (III) Ions Using Surface Functionalized Gold Nanoparticles

**DOI:** 10.3390/nano7100287

**Published:** 2017-09-22

**Authors:** Surendra Shinde, Dae-Young Kim, Rijuta Ganesh Saratale, Asad Syed, Fuad Ameen, Gajanan Ghodake

**Affiliations:** 1 College of Life Science and Biotechnology, Department of Biological and Environmental Science, Dongguk University-Seoul, Ilsandong-gu 10326, Goyang-si, Korea; shindesurendra9@gmail.com (S.S.); sbpkim@dongguk.edu (D.-Y.K.); 2 Research Institute of Biotechnology and Medical Converged Science, Dongguk University-Seoul, Ilsandong-gu 10326, Goyang-si, Korea; rijufbt@dongguk.edu; 3 Department of Botany and Microbiology, College of Science, King Saud University, P.O. 2455, Riyadh 11451, Saudi Arabia; assyed@ksu.edu.sa (A.S.); Fuadameen@ksu.edu.sa (F.A.)

**Keywords:** casein peptide, gold nanoparticles, aggregation, spectrophotometer, aluminum

## Abstract

A simple green route has been developed for the synthesis of casein peptide functionalized gold nanoparticles (AuNPs), in which casein peptide acts as a reducing as well as the stabilizing agent. In this report, AuNPs have been characterized on the basis of spectroscopic and microscopic results; which showed selective and sensitive response toward Al^3+^ in aqueous media, and Al^3+^ induces aggregation of AuNPs. The sensing study performed for Al^3+^ revealed that the color change from red to blue was due to a red-shift in the surface plasmon resonance (SPR) band and the formation of aggregated species of AuNPs. The calibration curve determines the detection limit (LOD) for Al^3+^ about 20 ppb (0.067 μM) is presented using both decrease and increase in absorbance at 530 and 700 nm, respectively. This value is considerably lower than the higher limit allowed for Al^3+^ in drinking water by the world health organization (WHO) (7.41 μM), representing enough sensitivity to protect water quality. The intensity of the red-shifted band increases with linear pattern upon the interaction with different concentrations of Al^3+^, thus the possibility of producing unstable AuNPs aggregates. The method is successfully used for the detection of Al^3+^ in water samples collected from various sources, human urine and ionic drink. The actual response time required for AuNPs is about 1 min, this probe also have several advantages, such as ease of synthesis, functionalization and its use, high sensitivity, and enabling on-site monitoring.

## 1. Introduction

Aluminum is a widely used metal in industry [1], a trivalent cation of aluminum (Al^3+^) can exist in both water and vegetables, thus it can move in the human body through food and water sources [2]. The average daily human exposure of Al^3+^ due to contamination is about 3 to 10 mg·kg^−1^ as stated by the WHO [3]. The major exposure source of the Al^3+^ in everyday life is from the usage of aluminum foil and containers, the risk of absorption of Al^3+^ ions by the human body is growing [4]. However, aluminum is not necessary for the human body, and its excessive exposure to the human body has an adverse effect, such as Parkinson’s disease [5]. Furthermore, the iron binding protein has the ability to carry Al^3+^ ions to the brain and damage the central nervous system [6]. Most prominently, the presence of Al^3+^ in the human brain in excessive amounts lead to the development of Alzheimers’s disease [7]. Thus, the detection of Al^3+^ using effective methods has drawn increasing interest in the areas of analytical, biological, environmental and medical sciences. For the same reason, several fluorescence-based sensing probes for different metal ions have been developed using organic dyes, however, these sensors showed moderate sensitivity and selectivity toward Al^3+^ [8,9]. In order to overcome the limitations of fluorophore-based sensing methods, a recent research has focused on building up highly sensitive methods suitable for analysis of water samples [10,11]. This research work puts effort towards developing highly water-soluble spectrophotometric probe for determination of Al^3+^ using casein peptide functionalized gold nanoparticles (AuNPs).

Changes in the optical properties of AuNPs can be easily monitored by the naked eye and aggregation-dependent spectral shifts [12]. Metal nanoparticles have the advantageous properties for developing sensing probe, including; a high aspect ratio, narrow size distribution, functionalized surface, and unique SPR band [13]. In particular, nanoparticles such as gold, silver, and copper having small size and unique localized SPR are useful for the development of optical devices [14,15]. In particular, AuNPs are widely used for fabrication of sensing platforms, due to their excellent optical and chemical properties [16,17]. Based on AuNPs, a number of sensing platforms have been developed for different metallic cations; however little attention has been devoted toward Al^3+^. This study demonstrates a green method for synthesis of AuNPs using casein peptides as reducing and stabilizing agent. The method enables incorporation of analyte binding casein peptide directly on AuNPs having the uniform shape and narrow size distribution, therefore allowing developing the spectrophotometric probe for Al^3+^. Importantly, the observed detection limit for Al^3+^ is 20 ppb which is significantly lower than the higher allowable limit for drinking water (200 ppb) as stated by the United States of America (U.S.) Environmental Protection Agency [18].

To the best of our knowledge, peptides have metal-complexing properties, hence we are interested in utilizing the casein peptides to form Al^3+^-peptide interactions. On the basis of the above consideration, we synthesized peptide-capped AuNPs have nanospherical morphology and small size employing hydrothermal reaction. AuNPs were able to recognize Al^3+^ ions with changes in color and spectral shifts of the SPR peak position in the aqueous medium. The AuNPs showed absorbance decrease at 530 nm in varying extent in an aqueous medium in the presence of common metal ions; however, Al^3+^ resulted in a significant spectral shift. The selectivity of Al^3+^ ion detection was achieved even in the presence of the other metal ions and mixture of metal ions. The moderately high sensitivity and spectral shift specifically at 700 nm in the presence of Al^3+^ ions is a clear signal of resilient and favored molecular interaction between the casein peptide and Al^3+^ ion. According to our knowledge, this could be where casein peptide is used for the selective sensing of Al^3+^ ions in aqueous medium. In this report, we introduced synthesis, characterization, and Al^3+^ sensing performance of AuNPs. These experimental results revealed that the aggregation process of AuNPs is dependent on the concentration of Al^3+^. Change in color from red to blue and unique spectral response of AuNPs was monitored by a portable spectrophotometer, showing practical applicability for on-site detection of Al^3+^.

## 2. Materials and Methods

### 2.1. Chemicals and Reagents

Casein acid hydrolysate (vitamin-free), HAuCl_4_ and NaCl were purchased from the Sigma Aldrich Chemicals (Yongin, South Korea). NaOH and HCl standard solution was purchased from the Dae Jung Chemicals (Shiheung, South Korea). Standard solution of the metallic cations includes, Al^3+^, Hg^2+^, Co^2+^, Cs^+^, As^+^, Cr^3+^, and Mn^2+^ were obtained from the Kanto Chemicals (Tokyo, Japan). Cu^2+^, Zn^2+^, Cd^2+^, and Pb^2+^ were obtained from the Nacalai Tesque Inc. (Kyoto, Japan). Stock solutions of the Al^3+^ (2.0 ppm) and (500 ppb) were prepared by the dilution of the standard solution (1000 ppm). 

### 2.2. Synthesis of the Gold Nanoparticles

The peptide-functionalized AuNPs were synthesized using vitamin-free casein peptide produced by acid hydrolysis as described previously [19]. Typically, casein peptide solution about 0.6% *w*/*v* was readily prepared in the distilled water and used freshly for the reduction of Au^III^ and stabilization of AuNPs. Briefly, 2 mL of 0.6% (*w*/*v*) peptide solution and 0.1 mL of NaOH (1 M) were mixed in 15.9 mL distilled water. 2 mL of HAuCl_4_ (10 mM) was added to preheated alkaline casein peptide mixture at 95 °C. The freshly prepared AuNPs were redispersed in distilled water after centrifugation at 9000 rpm for 15 min. Centrifuged AuNPs were free from the excess casein peptides, NaOH, and unreduced HAuCl_4_. The one-step synthesis process was successfully used to produce functionalized AuNPs, that are stable in aqueous media, and allows detection of Al^3+^ at room temperature.

### 2.3. Characterization of the AuNPs

The centrifuged AuNPs were stored in a refrigerator and used for characterization and sensing experiments. UV-vis absorption spectrum of the AuNPs was obtained by using (Optizen-2120 spectrophotometer, Daejeon, South Korea) in the wavelength range from 400 to 900 nm, with a resolution of 5 nm. The AuNPs imaging at nano-scale was performed using (Technai G^2^ F20 series, Ames, IA, USA) high-resolution transmission electron microscope (HR-TEM). These AuNPs samples were prepared by adding drops of nanoparticle solution on carbon coated copper grids and drying at room temperature. X-ray photoelectron spectroscopy (XPS) of AuNPs thin films was performed using (ULVAC-PHI Quntera SXM, Chigasaki, Japan) equipped with monochromatic Al Kα radiation. X-ray diffraction (XRD) was performed to test the crystalline nature of the AuNPs. The XRD spectra was recorded using Cu Kα_1_ radiation in the two theta range 30° to 80°. Attenuated total reflectance Fourier transform infrared (ATR-FTIR) spectroscopy (Thermo scientific Nicolet iS5, Waltham, MA, USA) was used for the characterization of surface chemistry and binding of the casein peptides on AuNPs surfaces.

### 2.4. Selectivity of the AuNPs

All the sensing experiments of AuNPs toward Al^3+^ were performed at room temperature (22 to 24 °C). The colorimetric and spectral response of AuNPs was revealed with environmentally relevant metal ions include, Co^2+^, Cu^2+^, Cd^2+^, Cr^3+^, Mn^2+^, Zn^2+^, Hg^2+^, Cs^+^, Pb^2+^, As^+^, and Al^3+^ at 200 ppb. Selectivity screening was carried out as follows: 600 μL of AuNPs solution was added to distilled water having particular metal ions and the total volume of the reaction solution was maintained about 1.0 mL. These suspensions were placed in the UV-vis spectrophotometer after 20 min of reaction and change in absorbance spectra were examined for these different metal ions. 

The UV-vis spectrum of AuNPs was also collected after reaction with one equivalent of Al^3+^ in the presence of two equivalent of the other metal ions include, Co^2+^, Cu^2+^, Cd^2+^, Cr^3+^, Mn^2+^, Zn^2+^, Hg^2+^, Cs^+^, Pb^2+^, and As^+^. Interference from individual metal ions was examined as follows: 600 μL of AuNPs solution was added to distilled water having particular metal ions. UV-vis absorbance spectra of AuNPs suspensions were collected after 24 h of incubation at room temperature. These suspensions were treated with target metal ion Al^3+^ and individual absorbance spectrums were collected within 20 min. 

### 2.5. Determination of the Al^3+^ Standard Solution

The measurement of the AuNPs sensitivity toward Al^3+^ was carried as follows: 400 μL of AuNPs solution was added to distilled water having particular metal ions at increasing concentrations of Al^3+^ (10 to 50 ppb). The absorbance spectra of the AuNPs suspensions having listed concentrations of Al^3+^ (0.0, 10, 20, 30, 40 and 50 ppb) were measured after 20 min of treatment. Quantitative detection of Al^3+^ was established repeatedly by observing an increase in absorbance intensity of AuNPs at 660 nm with respect to Al^3+^ concentration.

### 2.6. Effect of pH and Ionic Strength

Usually, the pH values and ionic strength of probe solution have the tremendous impact on the detection of target metal ions. The effect of diluted NaOH and HCl on AuNPs were investigated as follows: 600 μL of the AuNPs solution was added to distilled water, these suspensions were exposed to different molar concentrations of HCl and NaOH (0.0, 0.05, 0.1, 0.2, 0.3, 0.4 and 0.5 mM). These AuNPs suspensions were placed inside the UV-vis spectrophotometer and absorbance of the AuNPs was monitored after 5 min of incubation at 530 nm. Then, 90 μL of Al^3+^ (50 ppb) was added to each reaction mixture. The absorbance of these suspensions were again monitored after 1 and 5 min of incubation at 530 nm. The same protocol was performed to test the effect of the ionic strength on AuNPs dispersion and the sensitivity measured toward Al^3+^ (50 ppb) with different molar ratios of NaCl (0.0, 0.5, 1.0, 2.0, 3.0, 4.0 and 5.0 mM). 

### 2.7. Real-time Response of the AuNPs toward Al^3+^

The temporal decrease in absorbance of the SPR band of AuNPs was monitored at 530 nm. 700 μL of the AuNPs was added to distilled water and reacted with 50 ppb Al^3+^. Initially, absorbance was acquired up to 20 min with a 2 min of interval. The decrease in the absorbance was plotted against time. Afterwards, 400 μL of the AuNPs solution was added to distilled water. These suspensions were treated with different concentrations of Al^3+^ (10, 20, 30, 40 and 50 ppb). A real-time spectral response of AuNPs was recorded for the extended period of the incubation (20 to 420 min). 

### 2.8. Interference from other Metal Ions

High selectivity is an important factor of an excellent sensor. Therefore, the interference of a mixture of metal ions was investigated by observing the response of AuNPs to the mixture of metal ions under the same analytical conditions. These mixtures were having two equivalent more metal ions than that of the Al^3+^. Each mixture was prepared using three different metallic ions [(Mix A: Co^2+^, Cu^2+^, Cd^2+^) (Mix B: Cr^3+^, Mn^2+^, Zn^2+^), (Mix C: Hg^2+^, As^+^, and Pb^2+^)]. UV-vis absorbance spectra of the AuNPs in the presence of the metal ion mixtures were obtained after 5 min of incubation. Additional changes in the UV-vis spectrum of AuNPs treated with metal ion mixtures were also observed after reaction with 50 ppb Al^3+^. The fourth mixture was prepared by using 10 different environmentally relevant metal ions, Co^2+^, Cu^2+^, Cd^2+^, Cr^3+^, Mn^2+^, Zn^2+^, Hg^2+^, Cs^+^, Pb^2+^, and As^+^ to mimic the state of water. 600 µL of the AuNPs solution was added to the metal ion mixture and UV-vis spectrum of AuNPs was collected in the absence and presence of the target metal ion Al^3+^.

### 2.9. UV-vis Response toward Al^3+^ in Real Samples

After testing selectivity toward Al^3+^ and interference over common metallic cations practical application of the AuNP probe was revealed. Initially, the AuNP probe was applied to detect Al^3+^ in different water samples, urine, BSA, and ionic drink spiked with Al^3+^ (50 ppb). The monitoring spectral response of AuNPs toward Al^3+^ was carried out as follows: 400 μL of the AuNPs solution was added to respective samples. The absorbance spectra of the AuNPs suspension of AuNPs samples were measured after 20 min of incubation. These suspensions were exposed to Al^3+^ (50 ppb) and absorbance spectra of AuNPs samples were measured after additional 20 min of incubation. 

## 3. Results and Discussion

### 3.1. Synthesis of the AuNPs

**Figure 1:**
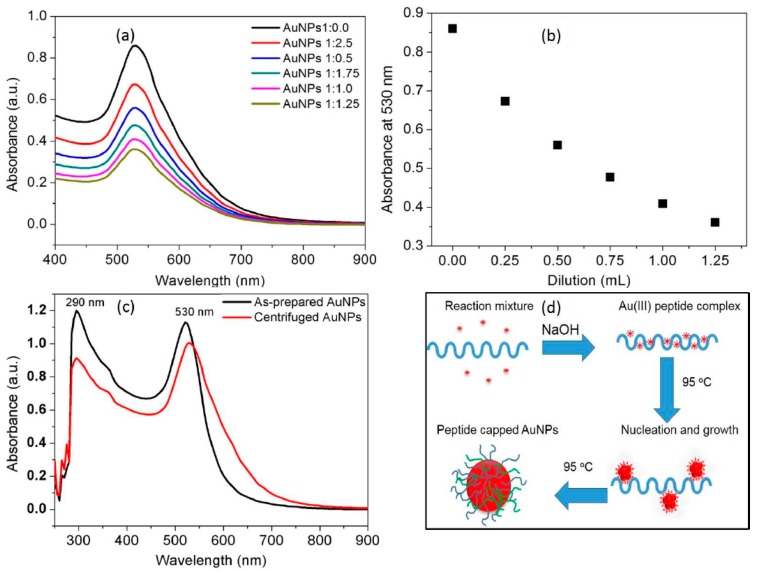
(**a**) Change in the plasmon absorption spectrum of the AuNPs under various dilution conditions by using distilled water; (**b**) A Beers and Lambert linear fit plotted from the experimental data collected at 530 nm under various dilution conditions by using distilled water; (**c**) The UV-vis spectrum of both as-prepared and centrifuged AuNPs in the UV range showing healthy capping of casein peptides on the AuNPs surfaces; (**d**) Schematic representation of casein peptide mediated synthesis and capping of the AuNPs.

In the present investigation, an unprecedented green process was used for the synthesis of the AuNPs by a simple treatment of gold salts with casein peptides. In the preliminary experiments, it was revealed that the reduction rate of Au ions and nucleation of AuNPs can be increased with elevated temperature conditions and in the presence of alkaline environment. Casein peptide mediated synthesis of AuNPs was carried out at 95 °C and it was noticed that the reduction rate of Au^III^ in turn improved by employing a deprotonated form of casein peptides. Thus, we resolve that the Au^III^ that reduced were consumed for the nucleation and growth of the nanocrystals by following classical nucleation and growth route as reported previously [20]. The absorbance maxima of AuNPs (530 nm), observed directly from the UV-vis spectra (Figure 1a), is in good agreement with the shape and narrow size-distribution as discussed in characterization section. A “bottom-up” approach used for the synthesis of small sized AuNPs from the properties of the casein peptide for HAuCl_4_ reduction and binding to AuNPs [21]. Peptides were used as a multifunctional reagent (reducing and capping agents) for the synthesis of biocompatible AuNPs [19], peptides isolated from casein protein was selected for its widely varying compositions and sequence [22].

A stable SPR band of AuNPs indicates suitability to develop sensing applications. Thus, it is vital to observe an effect of dilution on the plasmon resonance wavelength (λ_max_) and bandwidth (Δλ). The SPR band of AuNPs was examined under every successive addition of 0.25 mL of distilled water to 1.0 mL of AuNP suspension. It is significant to note that both λ_max_ and Δλ were sustained with the characteristic SPR band at low concentration ranges of AuNPs (Figure 1a). The absorption intensity at 530 nm was found to be linearly dependent on the dilution of AuNPs, indicates that casein peptide is able to protect AuNPs from aggregation and oxidation (Figure 1b). This result is in accordance with the Beer–Lambert law indicating excellent in vitro stability of AuNPs similarly to previous reports [23]. Thus, utilizing low concentration suspensions of AuNPs will be appropriate to develop selective and sensitive sensing probe for target metal ions. The synthesis method used for the production of AuNPs meets all 12 principles of the green chemistry, since no “man-made” chemicals, other than the Au^III^ were used. The possible binding of casein peptide to the AuNPs provides excellent in vitro stability in aqueous media, therefore these AuNPs are useful to employ their characteristic SPR band in sensing and biological applications [24,25]. 

Centrifugation of the AuNPs solution was executed to remove unbound casein peptide molecules and unreacted NaOH and HAuCl_4_ after synthesis. UV-vis spectra of the AuNPs solution before and after centrifugation are presented in Figure 1c. The peak appearing at 290 nm was also evident with centrifuged AuNPs, clearly, indicates the presence of the aromatic amino acid residues in the peptide sequence (Figure 1c). The UV-vis spectra of the AuNPs obtained before and after centrifugation shows minor changes in broadening and spectral shift due to the removal of the excess casein peptides (Figure 1c). Thus, the casein peptides were successfully used to functionalize AuNPs, without performing any special conjugation procedure. Consequently, centrifuged AuNPs were used to trigger effective coordination interactions with the target metal ions. Based on our understanding and literature reports, peptide ligand interactions on the AuNPs surface were mainly through hydrophobic side chains of amino acids and thiol functional groups [26]. Casein peptide capped AuNPs hybrids were illustrated in the schematic representation showing capping layer formed by strong conjugation and anchoring of peptide molecules on the surface of AuNPs (Figure 1d).

### 3.2. Characterization of the AuNPs

**Figure 2:**
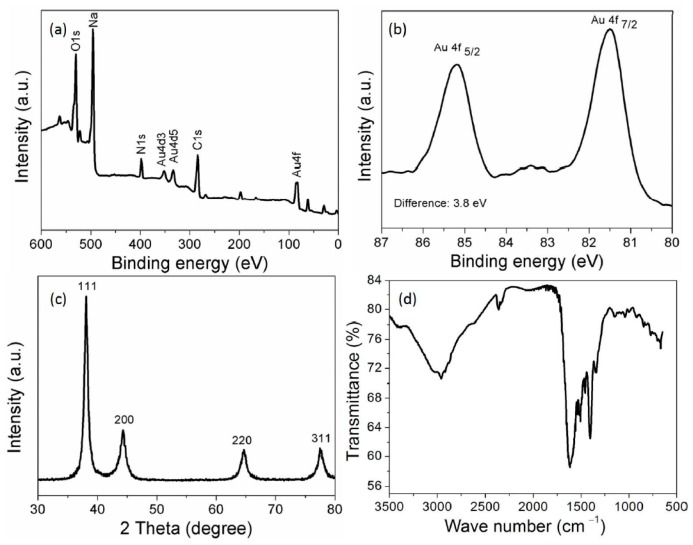
(**a**) XPS survey spectra of AuNPs; (**b**) Narrow scan spectra of AuNPs; (**c**) XRD spectra of AuNPs; (**d**) FTIR spectra of AuNPs.

**Figure 3:**
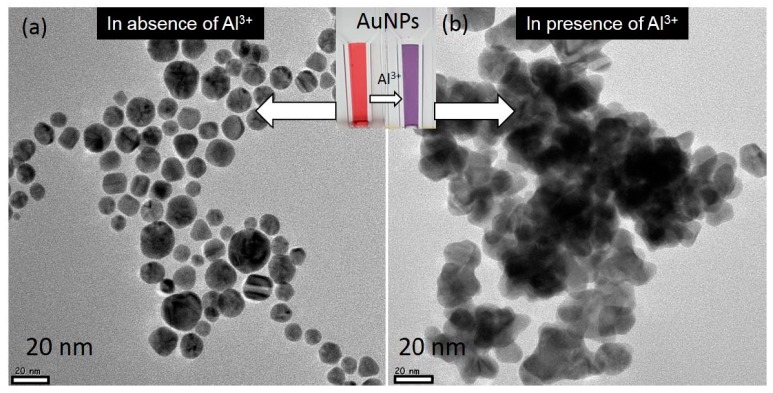
(**a**) The HR-TEM image of AuNPs in absence of Al^3+^; (**b**) The HR-TEM image of AuNPs in presence of Al^3+^. (Inset showing color of corresponding AuNPs solutions).

The survey scan of XPS spectra showed clear peaks for C 1s, N ls and O ls. These peaks confirm that AuNPs were capped with casein peptides (Figure 2a). The narrow scan of the XPS spectrum shows a doublet for AuNPs about at 85.1 and 81.5 eV allocated for the 4f_5/2_ and 4f_7/2_, respectively (Figure 2b). A spin-orbit splitting was about 3.8 eV, the representative for the reduction of Au^3+^ into Au was completed using casein peptides. The different diffraction peaks at (111), (200), (220), and (311) indicated that AuNPs were having a cubic structure (Figure 2c). The mean crystallite size of the AuNPs was found to be about 14 nm using Scherrer equation *D* = 0.94*λ*/*β*1/2 cos *θ*, seems in good agreement with the HR-TEM image. The ATR-FTIR bands correspond to the peptide were evident in the IR spectrum of the AuNPs obtained after centrifugation [27]. The characteristic IR peak found in the spectra at 1640 cm^−1^ corresponded to the amide I of carboxyl stretch vibration, indicating the successful binding of casein peptide molecules to AuNPs (Figure 2d). The characteristic peak of casein peptide skeleton vibration was shifted from 1550 to 1520 cm^−1^, which indicates benzene ring of the tyrosine and tryptophan were involved in the reduction of Au^III^ in addition to the other functional groups. The phenolic group of tyrosine can be involved in the reduction of Au^III^ ions into AuNPs, and it can be seem that the skeleton vibration of the benzene disappears when the phenolic groups play a role of reducing agent in the basic condition [28]. The FTIR spectra of casein peptide capped-AuNPs suggest that capping remains an integral part of AuNPs even after centrifugation, indicating successful conjugation of peptide molecules to the AuNPs, without using complicated conjugation protocols. The HR-TEM image suggests that AuNPs were having nanospheres morphology and small size despite the diverse amino acid residues in the peptide sequence. The HR-TEM image clearly showed that most of the AuNPs were well dispersed and have narrow size-distribution (5 to 20 nm) (Figure 3a).

### 3.3. Selectivity of the AuNPs toward Al^3+^

The experimental results suggest that casein peptide ligands on AuNPs provide excellent selectivity toward target metal ions Al^3+^. We tested whether AuNPs are capable of the binding to different metal ions and also the possibility in the development of the highly selective probe. The UV-vis spectra show the spectral response of the AuNPs toward various metal ions in aqueous media. The addition of 100 ppb Al^3+^ to the AuNPs solution resulted in a change in color from the red to dark blue within few seconds. The addition of the Al^3+^ also resulted a prominent red-shift towards the longer wavelength at 700 nm and decrease in absorbance of the SPR band at 530 nm. In contrast, other metal ions such as Cu^2+^, Co^2+^, As^+^, Cr^3+^, Zn^2+^, Cs^+^, Cd^2+^, Pb^2+^, Mn^2+^, and Hg^2+^ caused almost no red-shift and absorbance decrease. These results demonstrated that the synthesized AuNPs probe is highly selective for Al^3+^ over the tested metal ions (Figure S1a). Similar studies were reported for the functionalized nanoparticles by using nucleic acids, however, such methods are expensive and require complicated conjugation procedures [29,30]. Selective detection of the Al^3+^ in the presence of the other metal ions also resulted in consistent red-shifts and decrease in absorbance of the SPR band at 530 nm (Figure S1b). Thus, the excellent selectivity of the AuNPs is observed toward Al^3+^ with unique red-shift and consistent decrease in absorbance of the SPR band at 530 nm. In aqueous media, the affinity of Al^3+^ towards the functional carboxylic group of the casein peptide led to a noticeable aggregation and color change of casein peptide-AuNPs (Figure 3b). Aggregation has also been investigated by HR-TEM analysis: casein peptide-AuNPs images and their corresponding color was observed in the presence of Al^3+^ (Figure 3b), clearly shows that the change in the dispersion state and color occurs in the presence of Al^3+^. Metal binding affinity and selectivity was reported to form various structural complexes using both synthetic and natural peptides [31]. However, fairly few selective probes have been studied and reported for Al^3+^ [32]. Peptide-functionalized nanoparticles was also reported for the non-selective colorimetric response towards different metal ions such as Co^2+^, Hg^2+^, Pb^2+^, Pd^4+^, and Pt^2+^ [33,34,35]. 

### 3.4. Determination of the Standard Al^3+^ Concentration

**Figure 4:**
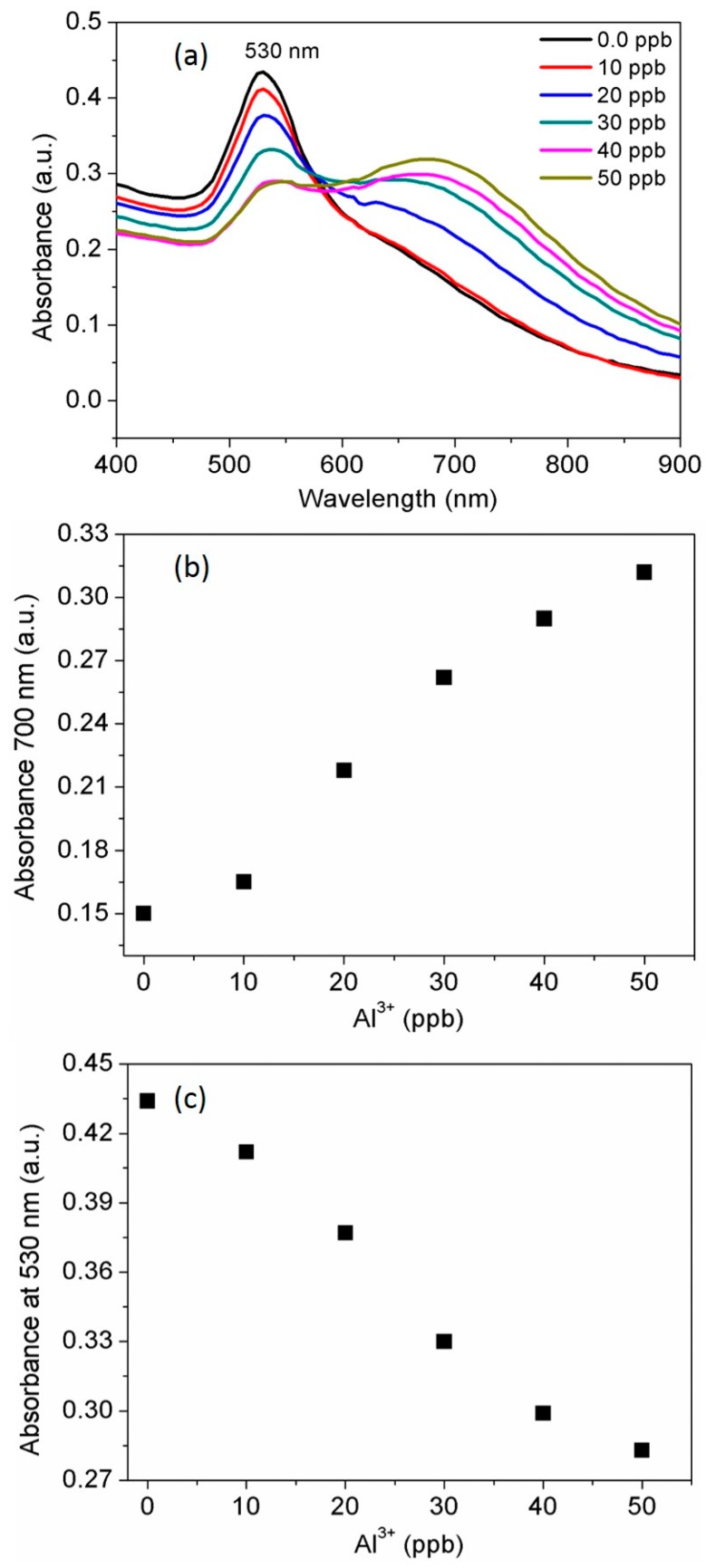
(**a**) The spectral response of AuNPs in the presence of increasing amounts of Al^3+^ at room temperature; (**b**) A linear fit curve of absorbance intensity at 700 nm vs. Al^3+^ concentration; (**c**) A linear fit curve of the absorbance intensity at 530 nm vs. Al^3+^ concentration.

After testing the selectivity of AuNPs probe toward Al^3+^ under identical experimental conditions, a target metal ion Al^3+^ was used for the detailed study. Al^3+^ concentration dependent red shift and increase in the absorbance intensity of AuNPs probe was detected at 700 nm. Then decrease in absorbance intensity of the SPR band at 530 nm is also discussed in this section, which indicates Al^3+^ dependent aggregation kinetics. The stability of the AuNPs probe gets inhibited upon complexation with Al^3+^. The absorption peak at 700 nm gradually increases its intensity as Al^3+^ concentration increased. The absorption spectra of the AuNPs was broadened and initiated to form red-shift immediately after the addition of 20 ppb Al^3+^ to the AuNPs solution. The broadening and red-shift of SPR bands were consistent with aggregation kinetics dictated by Al^3+^ concentration (Figure 4a). Moreover, complexation of the AuNPs ligands with Al^3+^ ions induces connectivity with neighboring AuNPs, thus resulting coordination complexes that allows to produce a chelation-dependent absorbance response which is found similar with the previous report [36]. Similarly, it was reported that 11-mercaptoundecanoic acid AuNPs could be induced to form aggregates in the presence of the Al^3+^ ions using chelation effect between the carboxyl groups [37]. Casein peptide functionalized AuNPs can be used for the sensitive detection of Al^3+^ ions in water with a linear range from 10 to 50 ppb and 20 ppb as a detection limit (LOD). Furthermore, absorbance at 700 nm vs concentration of the Al^3+^ was plotted for the quantitative detection (Figure 4b). Thus, AuNPs based probe was also demonstrated for quantitative detection of Al^3+^ by a monitoring decrease in absorbance of the SPR band at 530 nm (Figure 4c). The active chelation is involved and resulted enhanced absorbance at longitudinal band immediately after coordination of Al^3+^ with the amino acid residues in casein peptide sequence. As observed, AuNPs probe is appropriate for Al^3+^, its detection limit is considerably lower (20 ppb) than the higher limit allowed for Al^3+^ in drinking water by the WHO (7.41 μM) [38]. The high sensitivity of the probe is significantly important to establish selective and sensitive monitoring systems and to fulfill the growing need for analytical methods [39,40].

### 3.5. Effect of pH and Ionic Strength

**Figure 5:**
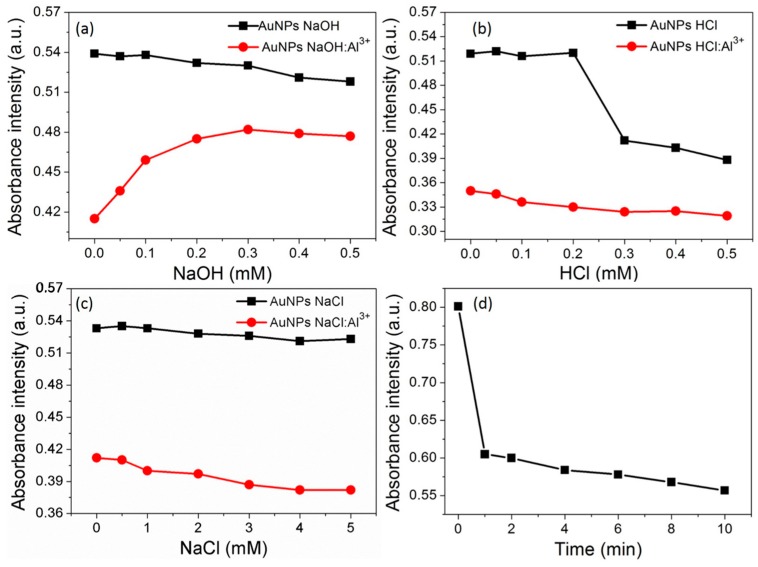
(**a**) The absorbance intensity of proposed nanosensor recorded at 530 nm in the absence and presence of the Al^3+^ at different molar ratio of NaOH; (**b**) The absorbance intensity of proposed nanosensor recorded at 530 nm in the absence and presence of the Al^3+^ at different molar ratio of HCl; (**c**) The effect of ionic strength on absorbance intensity recorded at 530 nm in the absence and presence of the Al^3+^; (**d**) The time course of the absorbance intensity of the AuNPs was recorded at 530 nm in the presence of the Al^3+^ (50 ppb) with 2 min interval.

Typically, pH values of AuNPs solution have the significant impact on the detection of the target analyte. Therefore, Al^3+^ sensing ability of the AuNPs at acidic and alkaline pH conditions were investigated. The results showed that AuNPs was stable within the range of 0.1 to 0.5 mM NaOH, and its response ability toward 50 ppb Al^3+^ was significantly increased within a neutral pH range (Figure 5a). The result showed that AuNPs were also stable within the range of 0.1 to 0.2 mM HCl, and UV-vis spectral response of the AuNPs toward Al^3+^ was enhanced within the range 0.1 to 0.5 mM HCl (Figure 5b). The results observed for the addition of the Al^3+^ with the increasing concentration of the HCl solution, indicating that the possibility aggregation contributed by both acidic pH and Al^3+^ coordination. For the rest of the experiments, distilled water was used as an analytical media for Al^3+^ without the addition of acid and/or alkali. 

The ionic strength is also an important parameter for the detection of the target analyte. The effect of the ionic strength of AuNPs stability is presented in Figure 5c, with increasing molar ratios of the NaCl. The absorbance intensity at 530 nm was not changed after the addition of NaCl solution, indicating the stability of AuNPs at different ionic strength. However, the addition of the Al^3+^ to AuNPs solution with increasing concentration of NaCl solution showed an enhanced analytical response at higher ionic strength.

### 3.6. Time Course of the AuNPs toward Al^3+^

The absorbance response of the SPR band at 530 nm was monitored after treating AuNPs with Al^3+^ (50 ppb). As shown in Figure 5d, the absorbance intensity of the AuNPs suspension decreases rapidly within 1 min of the incubation with Al^3+^. At first, the absorbance intensity was decreased to a minimum and then AuNPs probe was realized for rapid detection of Al^3+^ ions. As compared with the conventional detection methods, this probe enabled real-time detection with a response time of 5 s. Thus, casein peptide based AuNPs that are highly desirable to develop a real-time, portable, and a user-friendly analytical platform for rapid analysis of target metal Al^3+^ ions.

**Figure 6:**
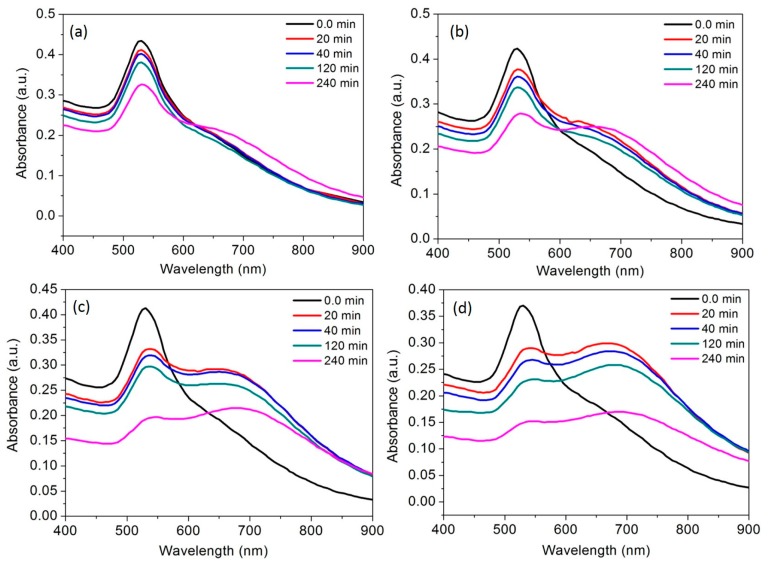
The time course of the spectral response of AuNPs at room temperature; (**a**) in the presence of the Al^3+^ (10 ppb); (**b**) in the presence of the Al^3+^ (20 ppb), (**c**) in the presence of the Al^3+^ (30 ppb); (**d**) in the presence of the Al^3+^ (40 ppb).

The recognition of Al^3+^ was directly linked to the casein peptide ligands those are electrostatically capped on the AuNPs surfaces. The UV-vis spectral response of AuNPs was observed with different concentrations of Al^3+^ ions (10, 20, 30 and 40 ppb) for the long period of a time (20 to 240 min). It was observed that the absorbance intensity of the SPR band and red-shifted longitudinal band consistently decreases with the increase in Al^3+^ concentration. A significant decrease in absorbance intensity without any shifts in the UV-vis spectra was observed after incubation of AuNPs with 10 ppb Al^3+^. This result suggests that there is a possibility of tuning a stable AuNPs aggregate using 10 ppb concentration Al^3+^ ions (Figure 6a). Similar absorbance profile of AuNPs allows a clear detection of Al^3+^ at 20 ppb with well-defined red-shift after a long period of incubation about 240 min (Figure 6b). After exposure of 30 and 40 ppb Al^3+^, a clear red-shift and consistent decrease in absorbance intensity was observed at both 530 and 700 nm, evidence suggests a transformation of stable AuNPs into unstable AuNPs aggregates (Figure 6c,d). Finally, these unstable aggregates formed at 30 and 40 ppb Al^3+^ were prone to settle down at the bottom of the tube. These results revealed that the coordination complex of peptide ligands and Al^3+^ ions continues to produce AuNPs aggregates due to the difference in electrostatic repulsion force. Thus, both the colorimetric and spectral response of the AuNPs are essential to understand coordination interactions and possibilities in the detection of target metal ions [41,42]. The UV-vis absorbance and/or fluorescence emission either decrease, quench, or enhance are the essential indicators to develop novel molecular sensing probes [43].

### 3.7. Plausible Coordination of Al^3+^ with Casein Peptide Ligands

The number of peptides/amino acid based assays has been reported for the detection of different metal ions [32,44]. The assay based on the metal ion induced aggregation of AuNPs in the presence of different amino acids and peptides that forms the complex with selective metal ions through their amine, carboxylic acid or side-chain functional groups have been reported elsewhere [44,45,46,47]. Charged, aromatic, and hydroxyl groups-containing amino acids are also well-known to interact with various metallic ions via noncovalent interactions [32]. The nitrogen atom of the amino group and oxygen atom of the carboxyl groups of Rhodamine is also reported for fluorometric detection of Al^3+^ ions [48,49]. Unfortunately, in these reports, application of inherent properties of the terminal carboxylic groups of the casein peptides is yet to be understood. As shown in Figure S2, for the initially well-dispersed AuNPs, upon the addition of Al^3+^ (50 ppb), the absorbance at 290 nm and 530 nm was decreased greatly and a new absorption band near to 700 nm appeared noticeably. Therefore, the decrease in absorbance at 290 nm in response to the aggregation of AuNPs was due to the complexation of Al^3+^ ions by aromatic amino acids present in casein peptide sequence. Accordingly, the color immediately turned from red to blue inset, these changes were considered to be attributed to the aggregation of AuNPs, which was also observed by HR-TEM images of Figure 3. Our experimental results suggest that casein peptide may be involved with Al^3+^ through hydrogen bond of N–H of the C-terminal amide group and CO of the N-terminal carboxyl group located at the end of a peptide. More importantly, this process is dose-dependent and manifests as a color change from red to blue. The previous results proved that casein peptide capped AuNPs signify a promising agent for selective recognition and coordinating Al^3+^ over other metal ions. A similar mechanism was reported to illustrate the affinity of Al^3+^ toward the carboxylic group of the pentapeptide that causes notable aggregation of the AuNPs and a clear color change from red to purple or blue [50,51]. A spectrophotometric probe based on casein peptide functionalized AuNPs exhibits high sensitivity and selectivity for detection of Al^3+^ ions in aqueous media. The LOD for AuNPs is calculated to be 20 ppb by the absorbance signals resulted in quantification of Al^3+^, which seems much lower than that of the reported literature values are summarized in Table 1. The binding sites for metal ions in the monolayer of AuNPs prepared with small organic molecules or biomolecules can serve as the recognition motif for developing chemosensing assays [25]. The casein peptides have few essential properties, such as excellent water-solubility, biocompatibility, and it has been used to prepare bioactive peptides for biological applications [52,53,54].

### 3.8. Interference from Other Metal Ions

**Figure 7:**
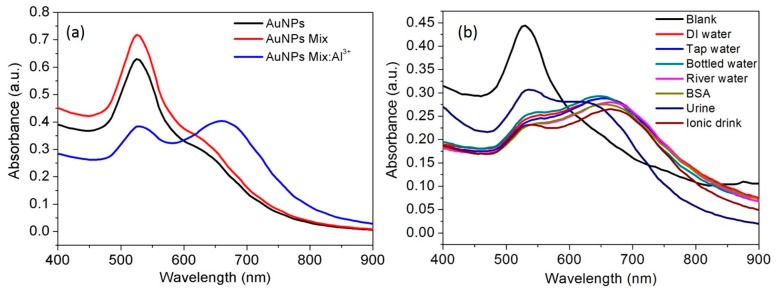
(**a**) The spectral response of AuNPs towards the Al^3+^ in presence of mixture of multiple kinds of metal ions at room temperature; (**b**) The spectral response of AuNPs towards the Al^3+^ in real samples at room temperature.

Less interference from other metal ions is significantly important to develop an excellent sensing probe. Therefore, interference from a mixture of metal ions was investigated by observing the spectral response of the AuNPs while detection of target analyte Al^3+^. The results showed that mixture of the metal ions could not show any colorimetric and spectral response (Figure S3a). However, Al^3+^ ions at 50 ppb can result unique spectral responses in corresponding metal ion mixtures (Figure S3b). Thus, the ability of casein peptide-AuNPs was demonstrated to recognize Al^3+^ in the presence of the mixture of environmentally relevant metal ions. No change in color was observed for 10 other metal ions (Co^2+^, Cu^2+^, Cd^2+^, Cr^3+^, Mn^2+^, Zn^2+^, Hg^2+^, Cs^+^, Pb^2+^, and As^+^) and their mixtures. The metal ion mixture treated with AuNPs solution did not affect the selectivity of the AuNPs toward Al^3+^ (Figure 7a). As understood from the interference results, AuNPs probe was able to detect target metal Al^3+^ ions in the presence 10 different metal ions, this indicates possibilities in developing a practical application for detection of target metal Al^3+^ ions. Similarly, luminescent assay reports that the isostructural lanthanide-organic compound can be used for sensitive and selective detection of Al^3+^ among various cations [55].

### 3.9. Spectral Response of Al^3+^ in Real Samples, Water, Urine and Ionic Drink

As real samples, water was collected from the tap in the laboratory, distilled water, bottle (commercial), river water, and human urine samples were spiked with a standard solution of Al^3+^ (50 ppb). In a typical method, identical amount of real samples spiked with Al^3+^ were was mixed with AuNPs solutions and mixed well and after that, the solution was used to observe spectral profiles. The Al^3+^ contents in the real samples were then directly measured by the casein peptide-AuNP, and the obtained results are presented in Figure 7b. The spectral response of casein peptide-AuNPs were examined with various real samples spiked with Al^3+^ (Figure 7b) and were used to compare with distilled water spiked with Al^3+^. The results obtained are identical with the added amount of Al^3+^ except human urine sample (Figure 7b), which validates the developed method for a vast range of real samples. The method, applied to estimate Al^3+^ in human urine, has shown dissimilar spectral profile due to interference from metabolites. The spectral response observed for the ionic drink is slightly stronger as compared with water samples and it may be due to the presence of various minerals. This report establishes the application of casein peptide-AuNPs as the recognition element for Al^3+^ ions present in real samples, thus, the proposed method is of academic and industrial interest. The spectral response induced by intraparticle plasmon-plasmon coupling can be examined by changes in SPR band, in addition to this it can be a basis for surface-enhanced Raman scattering of organic molecules capped on nanoparticles surfaces [56,57]. Al^3+^ spiked real samples was used to observe spectral shifts in a reasonable range (50 ppb) as compared with literature values summarized in Table 1 and reported for Al^3+^ detection using different approaches [48,58,59,60]. 

## 4. Conclusions

A simple green route has been used for the synthesis of casein peptide functionalized AuNPs. The casein peptide-AuNPs exhibited colorimetric and spectrophotometric detection of Al^3+^ in aqueous media with high sensitivity. Moreover, the Al^3+^ induced aggregation of casein peptide-AuNPs can be both stable and unstable depending on the concentration of Al^3+^. The sensitivity casein peptide-AuNPs for Al^3+^ is reasonably high (LOD: 20 ppb), as compared to the various literature reports and permissible level of Al^3+^ in drinking water as stated by WHO (7.41 μM). A mechanistic study showed that the detection of Al^3+^ was triggered by Al^3+^ ions that induced aggregation of casein peptide-AuNPs, and fruther leads to development of a red-shift of the SPR band causing an immediate color change from red to blue. Thus, casein peptide-AuNPs, can be used for detection of Al^3+^ in real water, urine, ionic drink samples, and the spectral results were compared with distilled water. The casein peptide-AuNPs showed high selectivity for Al^3+^ in the presence of the individual metal ions and the mixture of metal ions. Thus, the developed spectrophotometric probe was found suitable for making portable kits.

## Figures and Tables

**Table 1:** Literature values reported for the detection of Al^3+^ contents in a reasonable range using different colorimetric methods.

Method	Surface Chemistry	Limit of Detection (LOD) of Al^3+^	Solvent	Reference
Colorimetric	Citrate	1.0 μM	Water	[61]
Colorimetric	Pyridoxal derivative	0.51 μM	Water	[62]
Colorimetric	Ionic liquid	1.0 μM	Water	[63]
Colorimetric	Schiff base	0.29 μM	Water	[64]
Colorimetric	Polyacrylate	2.0 μM	Water	[65]
Spectral	Casein peptides	0.067 μM	Water	This work

## Supplementary Materials

The following are available online at http://www.mdpi.com/2079-4991/7/10/287/s1.

## References

[1] Mavhungu S.T., Akinlabi E.T., Onitiri M.A., Varachia F.M. (2017). Aluminum matrix composites for industrial use: advances and trends. Procedia Manuf..

[2] Ding W.-H., Cao W., Zheng X.-J., Fang D.-C., Wong W.-T., Jin L.-P. (2013). A Highly Selective fluorescent chemosensor for Al^III^ Ion and fluorescent species formed in the solution. Inorg. Chem..

[3] Valeur B., Leray I. (2000). Design principles of fluorescent molecular sensors for cation recognition. Coord. Chem. Rev..

[4] Tennakone K., Wickramanayake S., Fernando C.A.N. (1988). Aluminium contamination from fluoride assisted dissolution of metallic aluminium. Environ. Pollut..

[5] Vallejos S., Muñoz A., Ibeas S., Serna F., García F.C., García J.M. (2015). Forced solid-state interactions for the selective “Turn-On” fluorescence sensing of aluminum ions in water using a sensory polymer substrate. ACS Appl. Mater. Interfaces.

[6] Kim Y., Olivi L., Cheong J.H., Maertens A., Bressler J.P. (2007). Aluminum stimulates uptake of non-transferrin bound iron and transferrin bound iron in human glial cells. Toxicol. Appl. Pharm..

[7] Corain B., Bombi G.G., Tapparo A., Nicolini M., Zatta P., Perazzolo M., Favarato M. (1990). Alzheimer’s disease and aluminum toxicology. Environ. Health Perspect..

[8] Maity D., Govindaraju T. (2010). Pyrrolidine constrained bipyridyl-dansyl click fluoroionophore as selective Al^3+^ sensor. Chem. Commun..

[9] Upadhyay K.K., Kumar A. (2010). Pyrimidine based highly sensitive fluorescent receptor for Al^3+^ showing dual signalling mechanism. Org. Biomol. Chem..

[10] Dhara A., Jana A., Konar S., Ghatak S.K., Ray S., Das K., Bandyopadhyay A., Guchhait N., Kar S.K. (2013). A novel rhodamine-based colorimetric chemodosimeter for the rapid detection of Al^3+^ in aqueous methanol: fluorescent ‘OFF-ON’ mechanism. Tetrahedron Lett..

[11] Cui S., Tang Y., Lu R., Pu S. (2016). A multi-addressable diarylethene for the selective detection of Al^3+^ and the construction of a logic circuit. RSC Adv..

[12] Wang G., Sun W. (2006). Optical limiting of gold nanoparticle aggregates induced by electrolytes. J. Phys. Chem. B.

[13] Luo C., Wang Y., Li X., Jiang X., Gao P., Sun K., Zhou J., Zhang Z., Jiang Q. (2017). An optical sensor with polyaniline-gold hybrid nanostructures for monitoring pH in saliva. Nanomaterials.

[14] Lermé J., Bonnet C., Lebeault M.-A., Pellarin M., Cottancin E. (2017). Surface plasmon resonance damping in spheroidal metal particles: Quantum confinement, shape, and polarization dependences. J. Phys. Chem. C.

[15] Irshad M., Iqbal N., Mujahid A., Afzal A., Hussain T., Sharif A., Ahmad E., Athar M. (2013). Molecularly imprinted nanomaterials for sensor applications. Nanomaterials.

[16] Li Y., Cheng Y., Xu L., Du H., Zhang P., Wen Y., Zhang X. (2016). A Nanostructured SERS switch based on molecular beacon-controlled assembly of gold nanoparticles. Nanomaterials.

[17] Li Y., Schluesener H. J., Xu S. (2010). Gold nanoparticle-based biosensors. Gold Bull..

[18] Singha D.K., Mahata P. (2015). Highly selective and sensitive luminescence Turn-On-based sensing of Al^3+^ ions in aqueous medium using a MOF with free functional sites. Inorg. Chem..

[19] Ghodake G., Kim D.-Y., Jo J.H., Jang J., Lee D.S. (2016). One-step green synthesis of gold nanoparticles using casein hydrolytic peptides and their anti-cancer assessment using the DU145 cell line. J. Ind. Eng. Chem..

[20] Ji X., Song X., Li J., Bai Y., Yang W., Peng X. (2007). Size control of gold nanocrystals in citrate reduction: The third role of citrate. J. Am. Chem. Soc..

[21] Wang Z., Lévy R., Fernig D.G., Brust M. (2005). The peptide route to multifunctional gold nanoparticles. Bioconjug. Chem..

[22] Malin E.L., Alaimo M.H., Brown E.M., Aramini J.M., Germann M.W., Farrell H.M., McSweeney P.L.H., Fox P.F. (2001). Solution structures of casein peptides: NMR, FTIR, CD, and molecular modeling studies of αs1-casein, 1–23. J. Protein Chem..

[23] Shukla R., Nune S.K., Chanda N., Katti K., Mekapothula S., Kulkarni R.R., Welshons W.V., Kannan R., Katti K.V. (2008). Soybeans as a phytochemical reservoir for the production and stabilization of biocompatible gold nanoparticles. Small.

[24] Doty R.C., Tshikhudo T.R., Brust M., Fernig D.G. (2005). Extremely stable water-soluble Ag nanoparticles. Chem. Mater..

[25] Pezzato C., Maiti S., Chen J.L.Y., Cazzolaro A., Gobbo C., Prins L.J. (2015). Monolayer protected gold nanoparticles with metal-ion binding sites: Functional systems for chemosensing applications. Chem. Commun..

[26] Lévy R., Thanh N.T.K., Doty R.C., Hussain I., Nichols R.J., Schiffrin D.J., Brust M., Fernig D.G. (2004). Rational and combinatorial design of peptide capping ligands for gold nanoparticles. J. Am. Chem. Soc..

[27] Neda I., Vlazan P., Pop R.O., Sfarloaga P., Grozescu I., Segneanu A.-E., Krull I.S. (2012). Peptide and amino acids separation and identification from natural products. Analytical Chemistry.

[28] Selvakannan P.R., Swami A., Srisathiyanarayanan D., Shirude P.S., Pasricha R., Mandale A.B., Sastry M. (2004). Synthesis of aqueous Au core–Ag shell nanoparticles using tyrosine as a pH-dependent reducing agent and assembling phase-transferred silver nanoparticles at the air-water interface. Langmuir.

[29] Zhang X.-B., Kong R.-M., Lu Y. (2011). Metal ion sensors based on dnazymes and related DNA molecules. Annu. Rev. Anal. Chem..

[30] Xiang Y., Lu Y. (2014). DNA as sensors and imaging agents for metal ions. Inorg. Chem..

[31] Sovago I., Osz K. (2006). Metal ion selectivity of oligopeptides. Dalton Trans..

[32] Slocik J.M., Zabinski J.S., Phillips D.M., Naik R.R. (2008). Colorimetric response of peptide-functionalized gold nanoparticles to metal ions. Small.

[33] Xu W., Zhou Y., Huang D., Su M., Wang K., Hong M. (2014). A Highly sensitive and selective fluorescent sensor for detection of Al^3+^ using a europium(III) quinolinecarboxylate. Inorg. Chem..

[34] Chen Z., Sun Y., Zhang L., Sun D., Liu F., Meng Q., Wang R., Sun D. (2013). A tubular europium-organic framework exhibiting selective sensing of Fe^3+^ and Al^3+^ over mixed metal ions. Chem. Commun..

[35] Ghosh K., Majumdar A., Sarkar T. (2014). Selective sensing of Al^3+^ by naphthyridine coupled rhodamine chemosensors. RSC Adv..

[36] Hasni I., Yaakoubi H., Hamdani S., Tajmir-Riahi H.-A., Carpentier R. (2015). Mechanism of interaction of Al^3+^ with the proteins composition of photosystem II. PLoS ONE.

[37] Zhu R., Song J., Ma Q., Zhou Y., Yang J., Shuang S., Dong C. (2016). A colorimetric probe for the detection of aluminum ions based on 11-mercaptoundecanoic acid functionalized gold nanoparticles. Anal. Methods.

[38] Zhang M., Han J., Wu H., Wei Q., Xie G., Chen S., Gao S. (2016). Tb-MOF: A naked-eye and regenerable fluorescent probe for selective and quantitative detection of Fe^3+^ and Al^3+^ ions. RSC Adv..

[39] Guo L., Jackman J.A., Yang H.-H., Chen P., Cho N.-J., Kim D.-H. (2015). Strategies for enhancing the sensitivity of plasmonic nanosensors. Nano Today.

[40] Gómez-Graña S., Fernández-López C., Polavarapu L., Salmon J.-B., Leng J., Pastoriza-Santos I., Pérez-Juste J. (2015). Gold nanooctahedra with tunable size and microfluidic-induced 3D assembly for highly uniform SERS-active supercrystals. Chem. Mater..

[41] Lee J. T.Y., Leng Y., Chow K.L., Ren F., Ge X., Wang K., Lu X. (2011). Cell culture medium as an alternative to conventional simulated body fluid. Acta Biomater..

[42] Sato J.D., Kan M. (2001). Media for culture of mammalian cells. Current Protocols in Cell Biology.

[43] Kang K. A., Wang J., Jasinski J.B., Achilefu S. (2011). Fluorescence manipulation by gold nanoparticles: From complete quenching to extensive enhancement. J. Nanobiotechnol..

[44] Chen H., Zhang J., Liu X., Gao Y., Ye Z., Li G. (2014). Colorimetric copper(II) ion sensor based on the conformational change of peptide immobilized onto the surface of gold nanoparticles. Anal. Methods.

[45] Sener G., Uzun L., Denizli A. (2014). Colorimetric sensor array based on gold nanoparticles and amino acids for identification of toxic metal ions in water. ACS Appl. Mater. Interfaces.

[46] Li X., Wu Z., Zhou X., Hu J. (2017). Colorimetric response of peptide modified gold nanoparticles: An original assay for ultrasensitive silver detection. Biosens. Bioelectron..

[47] Zakaria H.M., Shah A., Konieczny M., Hoffmann J.A., Nijdam A.J., Reeves M.E. (2013). Small molecule- and amino acid-induced aggregation of gold nanoparticles. Langmuir.

[48] Manjunath R., Hrishikesan E., Kannan P. (2015). A selective colorimetric and fluorescent sensor for Al^3+^ ion and its application to cellular imaging. Spectrochim. Acta A.

[49] Ku K.-S., Muthukumar P., Angupillai S., Son Y.-A. (2016). A new rhodamine 6 G based chemosensor for trace level Al^3+^ and its thin film application in 100% aqueous medium. Sensor. Actuators B-Chem..

[50] Li X., Wang J., Sun L., Wang Z. (2010). Gold nanoparticle-based colorimetric assay for selective detection of aluminium cation on living cellular surfaces. Chem. Commun..

[51] Polavarapu L., Xu Q.-H. (2008). Water-soluble conjugated polymer-induced self-assembly of gold nanoparticles and its application to SERS. Langmuir.

[52] Phelan M., Aherne A., FitzGerald R.J., O’Brien N.M. (2009). Casein-derived bioactive peptides: Biological effects, industrial uses, safety aspects and regulatory status. Int. Dairy J..

[53] Nehete J.Y., Bhambar R.S., Narkhede M.R., Gawali S.R. (2013). Natural proteins: Sources, isolation, characterization and applications. Pharmacogn. Rev..

[54] Dallas D.C., Guerrero A., Parker E.A., Robinson R.C., Gan J., German J.B., Barile D., Lebrilla C.B. (2015). Current peptidomics: Applications, purification, identification, quantification, and functional analysis. Proteomics.

[55] Xu H., Fang M., Cao C.-S., Qiao W.-Z., Zhao B. (2016). Unique (3,4,10)-connected lanthanide–organic framework as a recyclable chemical sensor for detecting Al^3+^. Inorg. Chem..

[56] Qian X.M., Nie S.M. (2008). Single-molecule and single-nanoparticle SERS: From fundamental mechanisms to biomedical applications. Chem. Soc. Rev..

[57] Shiohara A., Langer J., Polavarapu L., Liz-Marzan L.M. (2014). Solution processed polydimethylsiloxane/gold nanostar flexible substrates for plasmonic sensing. Nanoscale.

[58] Yang N., Gao Y., Zhang Y., Shen Z., Wu A. (2014). A new rapid colorimetric detection method of Al^3+^ with high sensitivity and excellent selectivity based on a new mechanism of aggregation of smaller etched silver nanoparticles. Talanta.

[59] Alam R., Mistri T., Bhowmick R., Katarkar A., Chaudhuri K., Ali M. (2015). Dual channel selective fluorescent detection of Al^3+^ and PPI in mixed aqueous media: DFT studies and cell imaging applications. RSC Adv..

[60] Sinha S., Chowdhury B., Ghosh P. (2016). A Highly sensitive ESIPT-based ratiometric fluorescence sensor for selective detection of Al^3+^. Inorg. Chem..

[61] Chen S., Fang Y.-M., Xiao Q., Li J., Li S.-B., Chen H.-J., Sun J.-J., Yang H.-H. (2012). Rapid visual detection of aluminium ion using citrate capped gold nanoparticles. Analyst.

[62] Bothra S., Kumar R., Sahoo S.K. (2015). Pyridoxal derivative functionalized gold nanoparticles for colorimetric determination of zinc(II) and aluminium(III). RSC Adv..

[63] Chen W., Jia Y., Feng Y., Zheng W., Wang Z., Jiang X. (2015). Colorimetric detection of Al(III) in vermicelli samples based on ionic liquid group coated gold nanoparticles. RSC Adv..

[64] Huang P., Li J., Liu X., Wu F. (2016). Colorimetric determination of aluminum(III) based on the aggregation of Schiff base-functionalized gold nanoparticles. Microchim. Acta.

[65] Kumar A., Bhatt M., Vyas G., Bhatt S., Paul P. (2017). Sunlight induced preparation of functionalized gold nanoparticles as recyclable colorimetric dual sensor for aluminum and fluoride in water. ACS Appl. Mater. Interfaces.

