# Supplementary material for: A Spectral Probe for Detection of Aluminum (III) Ions Using Surface Functionalized Gold Nanoparticles
Source: Nanomaterials (Basel). 2017 Sep 22;7(10):287. doi: 10.3390/nano7100287 (PMC5666452; doi:10.3390/nano7100287)
Supplement: Supplementary file 1 [file nanomaterials-07-00287-s001.docx]

**Electronic Supplementary Information:**

**A spectral probe for detection of aluminum (III) ions using surface functionalized gold nanoparticles**

Surendra Shinde ^a^, Dae-Young Kim ^a^, Rijuta Ganesh Saratale ^b^, Asad Syed ^c^, Fuad Ameen ^c^, and Gajanan Ghodake ^a,^*

^a^ Dongguk University-Seoul, College of Life Science and Biotechnology, Department of Biological and Environmental Science, Ilsandong-gu 10326, Goyang-si, South Korea; shindesurendra9@gmail.com (S.S.); sbpkim@dongguk.edu (D.-Y.K.)

^b^ Research Institute of Biotechnology and Medical Converged Science, Dongguk University-Seoul, Ilsandong-gu 10326, Goyang-si, Republic of Korea,rijufbt@dongguk.edu (R.G.S.)

^c^ Department of Botany and Microbiology, College of Science, King Saud University, P.O. 2455, Riyadh 11451, Saudi Arabia, assyed@ksu.edu.sa (A.S.); Fuadameen@ksu.edu.sa (F.A.)

* Correspondence: ghodakegs@gmail.com (G.G.); Tel.: +82-31-961-5159; Fax: +82-31961-5122


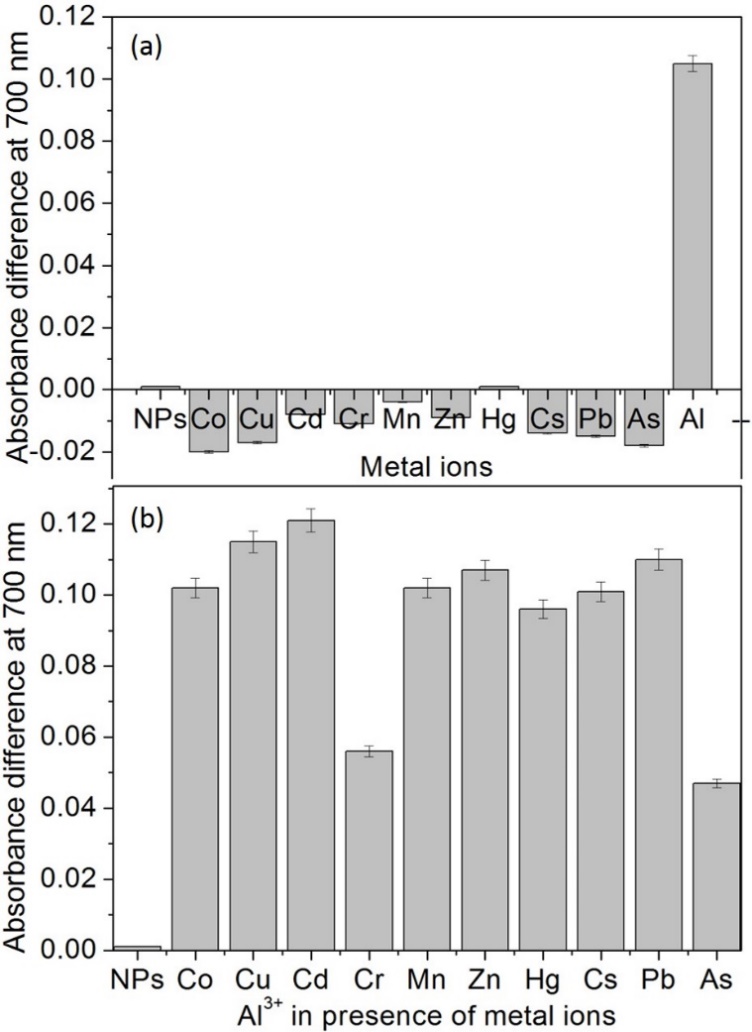


**Figure S1.** (**a**) The absorbance response of AuNPs recorded at 700 nm after treatment with different metal ions as labelled in the figure; (**b**) The absorbance response of AuNPs recorded at 700 nm after treatment with 1 equivalent of the Al^3+^ to the solution containing 2 equivalent of other metal ions.


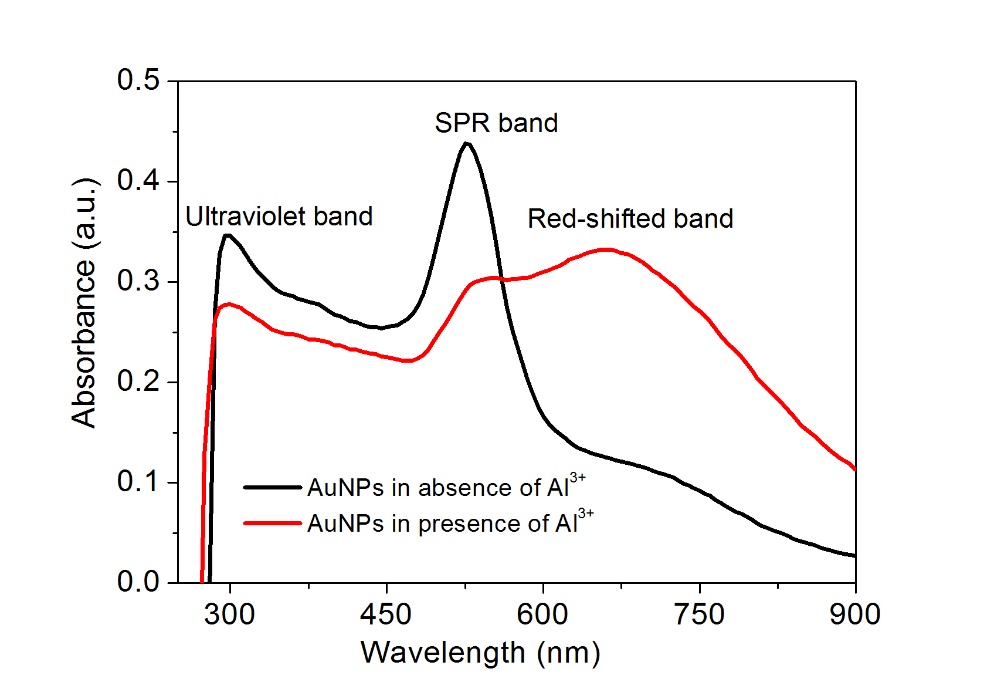


**Figure S2.** UV-vis spectra of casein peptide capped AuNPs in absence and presence of Al^3+^, in the range from 250 to 950 nm showing three different peaks.


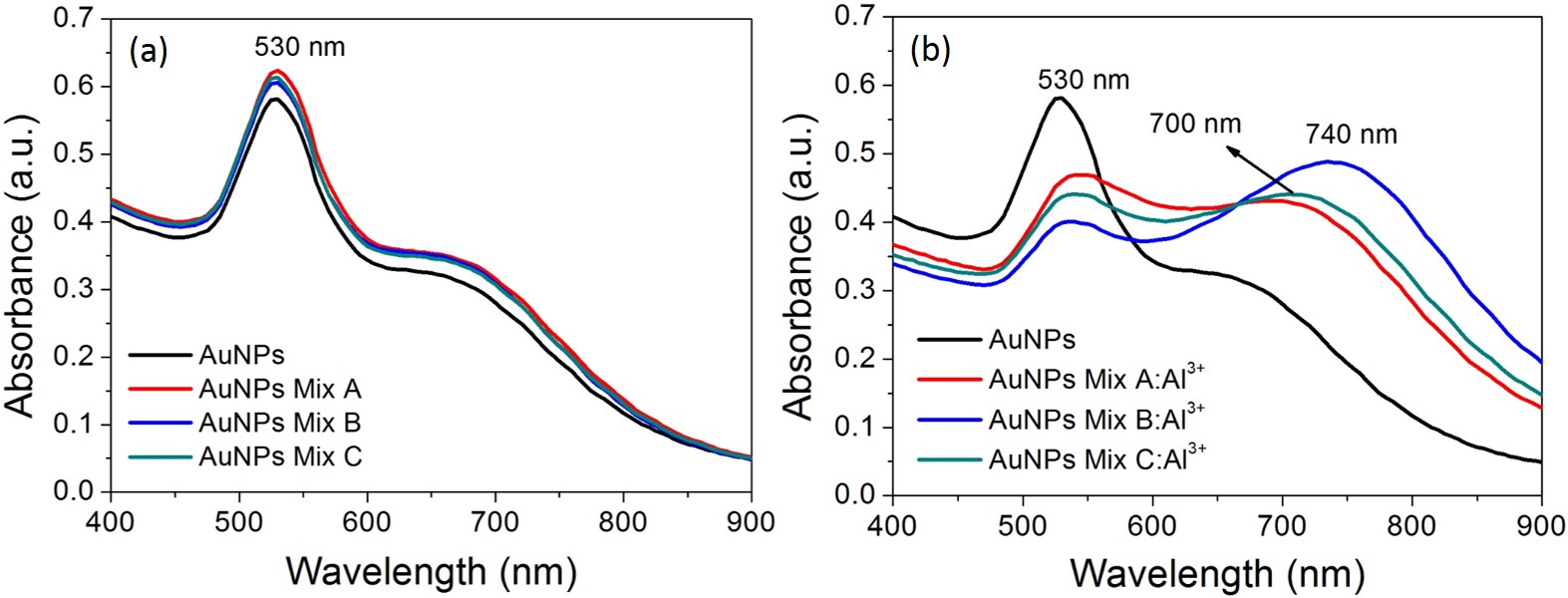


**Figure S3**. (**a**) The spectral response of AuNPs with the mixture A, B and C in absence of the Al^3+^ at ambient temperature; (**b**) The spectral response of AuNPs with the mixture A, B, and C in presence of the Al^3+^ at ambient temperature.
